# Distance Between Players During a Soccer Match: The Influence of Player Position

**DOI:** 10.3389/fpsyg.2021.723414

**Published:** 2021-08-19

**Authors:** David Garrido, Daniel R. Antequera, Roberto López Del Campo, Ricardo Resta, Javier M. Buldú

**Affiliations:** ^1^Laboratory of Biological Networks, Centre for Biomedical Technology, Universidad Politécnica de Madrid (UPM), Madrid, Spain; ^2^Complex Systems Group and GISC, Universidad Rey Juan Carlos, Móstoles, Spain; ^3^Mediacoach—LaLiga, Madrid, Spain

**Keywords:** soccer, player movement, distance, player position, modelling

## Abstract

In this study, we analyse the proximity between professional players during a soccer match. Specifically, we are concerned about the time a player remains at a distance to a rival that is closer than 2 m, which has a series of consequences, from the risk of contagion during a soccer match to the understanding of the tactical performance of players during the attacking/defensive phases. Departing from a dataset containing the Euclidean positions of all players during 60 matches of the Spanish national league (30 from *LaLiga Santander* and 30 from *LaLiga Smartbank*, respectively, the first and second divisions), we analysed 1,670 participations of elite soccer players. Our results show a high heterogeneity of both the player-player interaction time (from 0 to 14 min) and the aggregated time with all opponents (from <1 to 44 min). Furthermore, when the player position is taken into account, we observe that goalkeepers are the players with the lowest exposure (lower than 1 min), while forwards are the players with the highest values of the accumulated time (~21 min). In this regard, defender-forward interactions are the most frequent. To the best of our knowledge, this is the largest dataset describing the proximity between soccer players. Therefore, we believe these results may be crucial to the development of epidemiological models aiming the predict the risk of contagion between players and, furthermore, to understand better the statistics of all actions that involve proximity between players.

## Introduction

The recent ability to detect, record, and analyse all players' positions during a soccer match has boosted a diversity of studies about player's technical, tactical and physical performance (Gudmundsson and Horton, 2017). The majority of these studies focused on the performance of players based on distance (Brito Souza et al., 2020; Clemente et al., 2020), velocity (Marcelino et al., 2020) or acceleration (Dalen et al., 2016) at individual level. Only recently, several new metrics evaluating tactical aspects of soccer teams have been designed on the basis that players interact between them (Taki and Hasegawa, 2000; Link et al., 2016; Spearman et al., 2017; Fernandez and Bornn, 2018), introducing a point of view based on the paradigm of Complexity Sciences (Buldú et al., 2018). However, the fact that tracking datasets are quite difficult to be obtained and, sometimes, inaccessible due to the lack of tactical cameras in some stadiums, has hindered the understanding of how players interact between them. With this regard, there is a need of studies analysing of the distance between players distributes, which is crucial to understand the collective behaviours in sports. In this paper, we have investigated the amount of time that soccer players are closer than a threshold distance to other players. Thanks to the Mediacoach system (Mediacoach, 2020) installed at all stadiums of the Spanish first and second divisions, we had access to the positions of all players with an accuracy of <10 cm, which allowed us to extract the distance between players at every moment of the match. The motivation behind our study is two-fold. On the one hand, the knowledge of distancing between players is the starting point to developing epidemiological models in the context of sports competitions. Social distancing has been identified as one of the main variables affecting virus transmission both in close and open spaces (Wang et al., 2020). Despite the probability of outdoor transmission is estimated to be much lower than indoors (Nishiura et al., 2020), there is evidence of outdoor infections of SARS-CoV-2, as it is the case of influenza or some adenovirus (Bulfone et al., 2021). With this regard, in reference (Knudsen et al., 2020), the authors developed a model evaluating the risk of being infected by SARS-CoV-2 combining the distancing between players with the probability of stepping a region of the field where another player was placed 2 sec before. The analysis of 14 matches of the Danish national league showed that the average exposure time of a player during a soccer match was below one and a half minutes. Recently, Gonçalves et al. (2020) developed a more complete model where the distancing between players was combined by the respiratory exposure of players, the latter based on the movement of respiratory droplets. The analysis of 2 matches of the Portuguese national league reported a maximum contact time per pair of individuals of six and a half minutes, with an average time close to 30 sec. Note that studies designing epidemiological models in the context of sports competitions, such as (Buldú et al., 2020a), require for an accurate characterisation of the contact times between players.

On the other hand, the distancing between players can also be related to the tactical performance of soccer teams (Memmert et al., 2017). The player-player coordination is crucial to understand the performance of the tactical guidelines during a given match. Distancing between players, together with their speed and acceleration, is a key variable to understand their coordination. As shown by Marcelino et al. (2020), the spatial location of players and their role in the team is related to the coordination of their movements and, furthermore, most successful teams show a higher player-player coordination. More recently, in reference (Buldú et al., 2020b), the authors proposed constructing signed proximity networks that captured the spatial organisation of teams during the defensive and attacking phases. These networks take into account the distances with teammates and rivals to create, respectively, positive and negative links between them, which evolve along with the match. At the team level, distancing between players also influences team formations. Several positional indices have been defined to describe the location of a team on the pitch, such as the team centroid, the stretch index or the convex hull area (Castellano et al., 2017; Clemente et al., 2018). All these metrics, especially the dispersion of the players location around the team centroid, are strongly influenced by the distancing between players. At the same time, the automatic detection of team formations is another practical application where player-player distancing is crucial. Machine learning algorithms are currently used to detect the organisation of soccer teams and their relationship with classical team formations, such as 4-4-2 or 5-3-2 (Wu et al., 2018). However, the fact that players' positions overlap during a match makes the assessment of player distancing to be crucial in order to design successful algorithms (Bialkowski et al., 2014). In view of all, the aim of this paper is quantifying what are the expected distances between players during a football match and how the role of a player in the team influences the distancing to the rest of the players. We analysed the tracking datasets of 60 soccer matches of the Spanish national leagues (LaLiga Santander and LaLiga Smartbank). As far as we are aware, this is the largest dataset concerning the analysis of the proximity between soccer players, since references (Gonçalves et al., 2020) and (Knudsen et al., 2020) comprised 2 and 14 matches, respectively. We restricted our analysis to the distance between rival players, since one of the main applications of our study is assessing the risk that SARS-CoV-2 is transmitted from one team to another. Importantly, we focused on the interplay between the player position and the time accumulated with a rival closer than a threshold distance. As we will see, we report a high heterogeneity in the proximity between players that, in turn, is constrained by the position and type of interaction.

## Materials and Methods

### Study Design and Settings

We analysed the distancing between football players during a match by (i) recording their locations and (ii) measuring the time players where closer than a threshold distance *D* of either an opponent or a teammate. The datasets consisted of the tracking of the position of *N* = *1,670* participations of elite soccer players during *L* = *60* matches of the first and second division of the Spanish national league (LaLiga Santander and LaLiga Smartbank, respectively), all of them obtained during the 2018/2019 season, specifically fixtures 14 and 15 of both competitions. Datasets were supplied by *LaLiga* software *Mediacoach*^®^ (Mediacoach, 2020). A multi-camera tracking system recorded each player's position on the pitch with a sampling rate of Δ*f* = *25* frames/second, using a stereo multi-camera system composed of two units placed at either side of the midfield line (*Tracab Optical Tracking System*) (Linke et al., 2020). Each multi-camera unit contained three cameras with a resolution of 1,920 ×1,080 pixels that were synchronised to provide a panoramic picture and created the stereoscopic view for triangulating the players and the ball. An experienced operator corrected the position of players in the case of a temporal loss of any location. Importantly, datasets obtained by the *Mediacoach*^®^ system have been previously validated with GPS (Felipe et al., 2019; Pons et al., 2019).

### Participants and Bias

Each player was tagged with his position in the team, which has been split into five categories: goalkeepers *(N*_1_ = *120)*, defenders *(N*_2_ = *471)*, midfielders *(N*_3_ = *520)*, forwards *(N*_4_ = *209)* and substitutes *(N*_5_ = *350)*.Note that the latter category did not explicitly contain the position of players, however, we decided to maintain it in this form since, as we will see, the fact that a player did not participate since the beginning of a match drastically constraints the contact time with the rival players. Also, note that *N*_*i*_ refers to the total number of participations tagged with the corresponding position. Since datasets contained two fixtures, the same player could have contributed more than one time to *N*_*i*_. In other words, each match has at least 22 contributions (11 per team), plus the number of substitutes of each team.

### Variables and Statistical Methods

We developed an algorithm to evaluate the distance between players with Matlab®. At each time step, the distance *d*_*ij*_ between every pair of players *i* and *j* was determined using the tracking datasets, which contained the Euclidean position of all players. We set a threshold distance between players of *D* = *2* m and counted the number of frames *f*(*i, j*) each pair of players had been closer (or equal) than the distance *D*. Finally, the total time accumulated by each pair of players was obtained as Δ*t*_*accum*_(*i, j*) = Δ*t f*(*i, j*), where *t* = 0.04 *s* was the time step between two consecutive frames (i.e., the inverse of the sampling rate). We call this variable the player-player interaction time. Next, calculate the aggregated time *t*_*aggr*_(*i*), which is obtained by adding the time accumulated of player *i* to all his rivals $t_{aggr}\left(i\right)=\underset{j}{\sum }t_{accum}\left(i,j\right)$.

### Statistical Analysis

The analysis includes the calculation of the average values of the player-player contact time and the aggregated time to all players. For each average value we also obtain the standard deviation, which corresponds to the error bars plotter in the figures.

## Results

**Figure 1:**
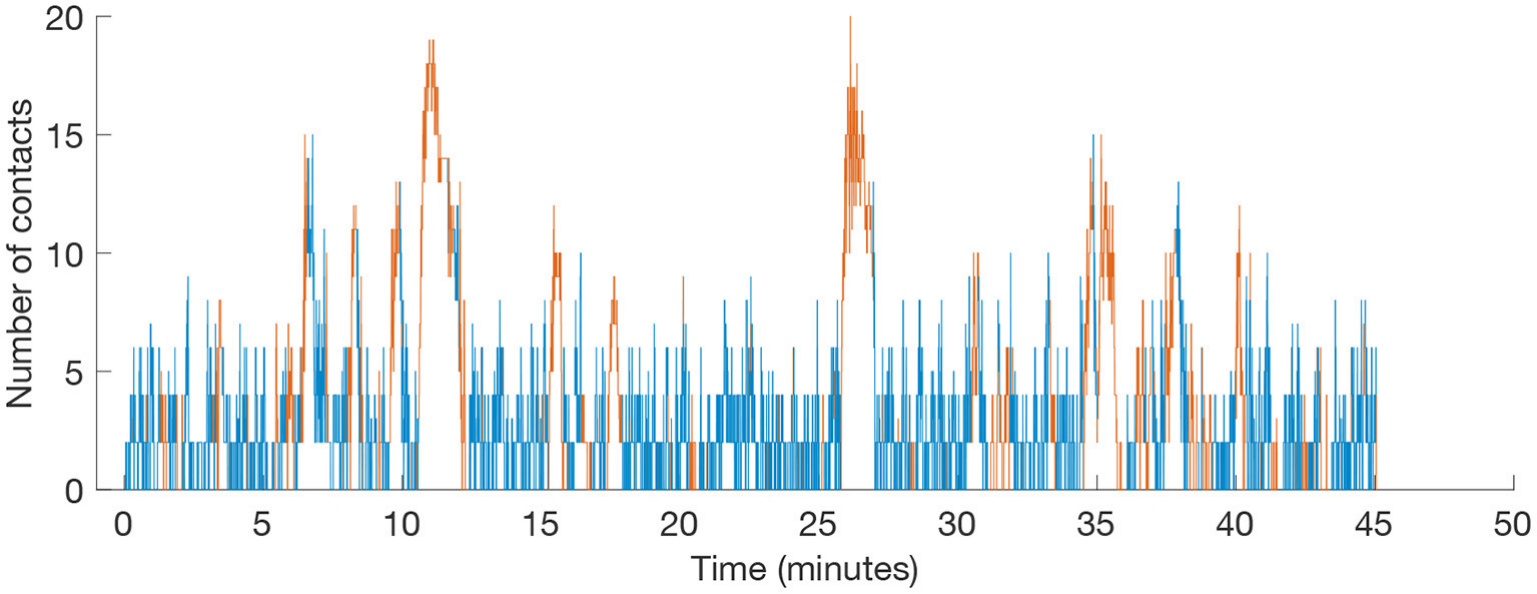
Proximity between players. For a given match played at LaLiga Santander during season 2018/2019, we count how many players are closer than a threshold distance *D*. In this particular example, *D* = *2* m. Blue and red colours indicate, respectively, whether the ball is being played or the match is stopped. The highest peaks correspond to fouls (minutes 11 and 26), corners (minutes 6) and the celebration of a goal (minutes 35).

**Figure 2:**
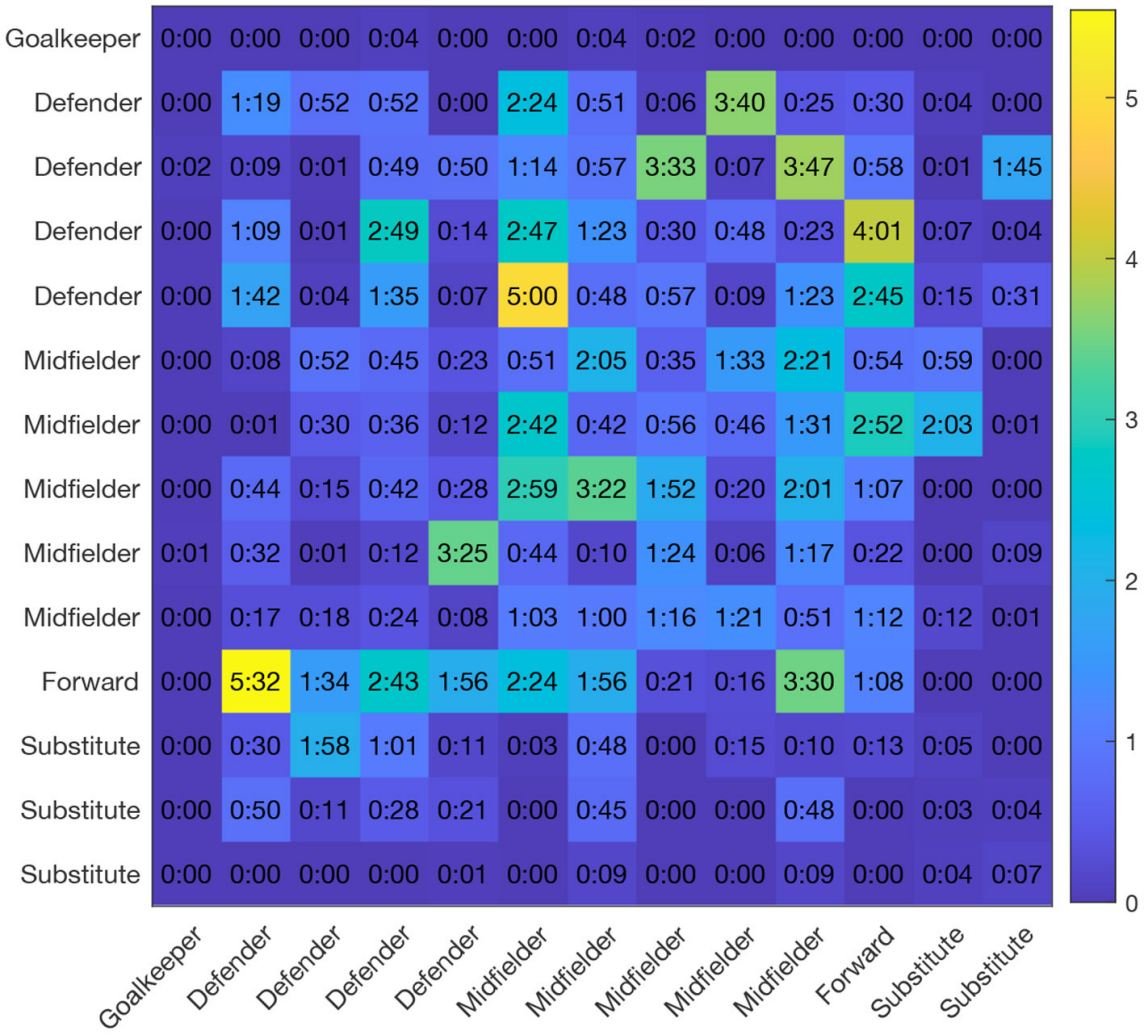
Accumulated time with the rival players $t_{accum}^{rivals}\left(i,j\right)$. For the same match of Figure 1, we counted the time each player had been closer than *D* = *2* m to an opponent. Elements of the matrix indicate min and sec. Each player was tagged according to his position in the team: (1) goalkeeper, (2) defender, (3) midfielder, (4) forward, and (5) substitute. We can observe how the time in the risk zone is quite heterogeneous, ranging from zero sec to five and a half min.

Far from being trivial, the proximity between players evolves during a match and its fluctuations are strongly influenced by the context of the game, as we can see in Figure 1. In this example, we can see how the total number of players that are closer than a distance *D* to any other player remains below 5 during most of the time. However, there are situations where the number of contacts increases, leading to high peaks with a very high number of players. In the particular example of Figure 1, the peaks correspond to fouls, corners and goal celebrations. As a consequence, each player accumulates a certain time in close contact to another player. Figure 2 shows a real example (same match as in Figure 1) of the matrix of accumulated time between rivals $t_{accum}^{rivals}\left(i,j\right)$ during the 90 min of a match.

**Figure 3:**
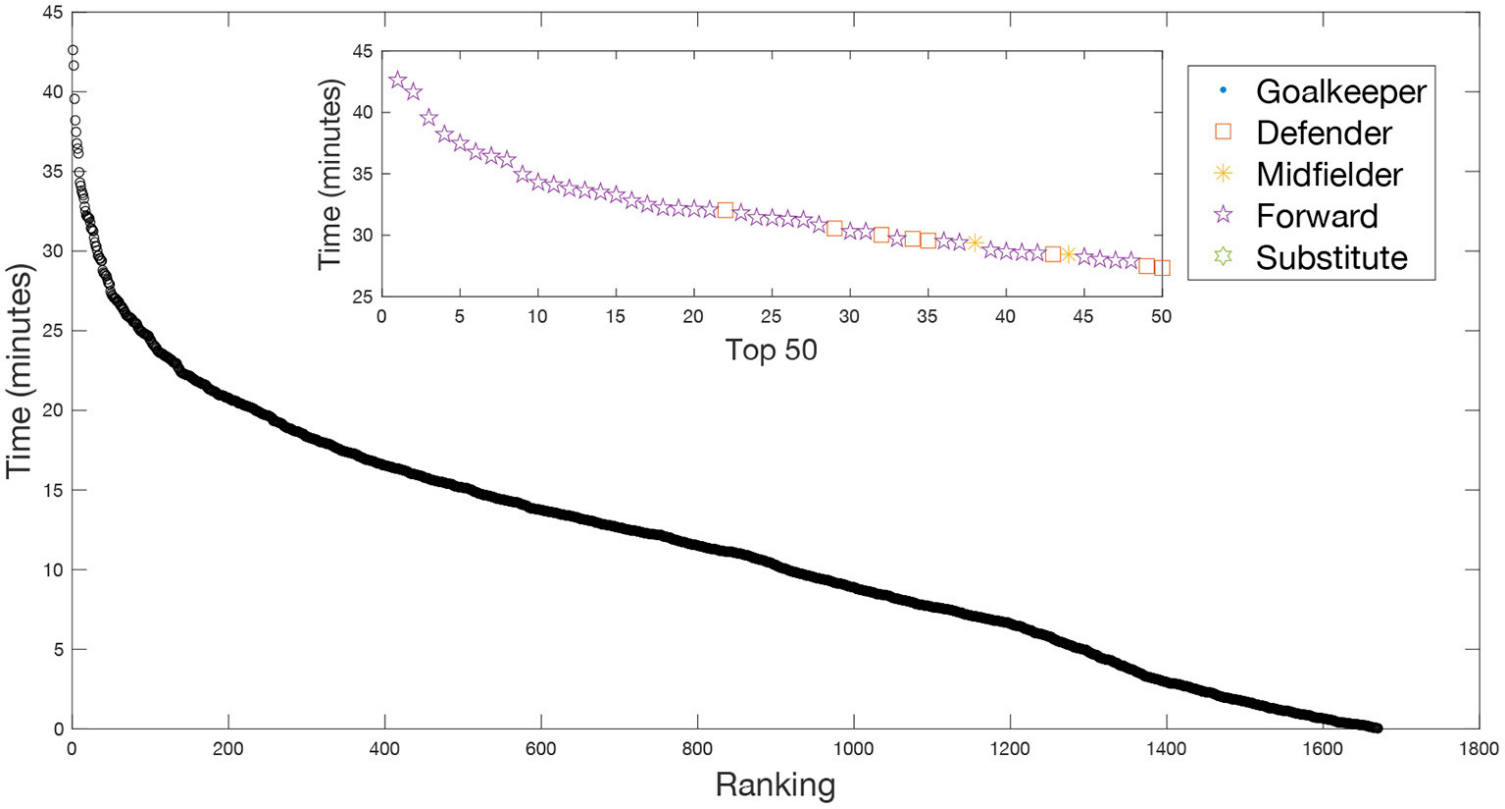
Heterogeneity of the contact time. Ranking of the aggregated time *t*_*aggr*_ of each player from all his rivals during a whole match. Each point accounts for the total time a player has been with at least one opponent closer than a distance *D* = *2* m. The inset shows the aggregated time of players at the top 50. We can observe that the ranking is leaded by forwards. Only eight defenders and two midfielders appear in the top 50, all of them beyond the 20th position.

In Figure 3 we plot the ranking distribution of the total time accumulated by each player during the whole match with any of the rival players at a distance closer (or equal) to *D* = *2* m. Note that we plot the aggregated time from all the rival players *t*_*aggr*_(*i*), and not the individual ones (also called player-player interactions). We obtained an average aggregated time for all players |*t*_*aggr*_| = 11. 54 min, however, we can observe in Figure 3 that there is a high heterogeneity in the ranking distribution. Consequently, there is a small group of players whose aggregated time is much higher than the average. The inset of Figure 3 shows a zoom of the main figure, including only the 50 players with the highest aggregated times with their corresponding positions (see figure caption).

Interestingly, we can see that most of these players are forwards, suggesting that the player position strongly influences the aggregated time. In fact, the top 20 is only occupied by forwards, and only eight defenders and two midfielders appear in the top 50.

**Figure 4:**
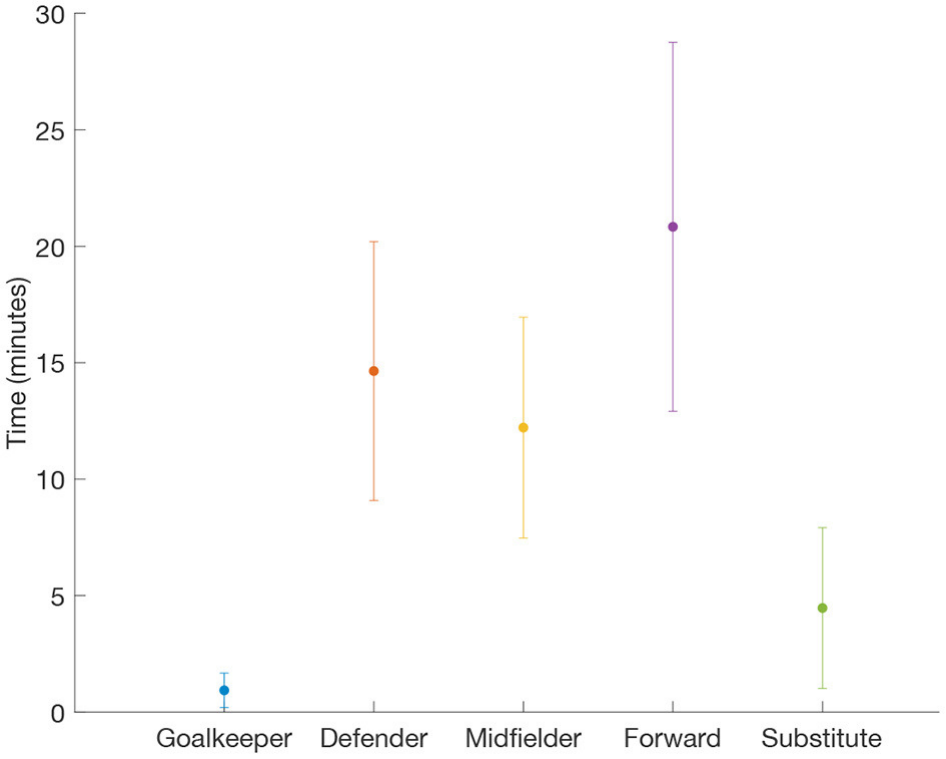
Aggregated time by player position. Average time |*t*_*aggr*_| that a player, according to his position, has accumulated with at least one rival at a distance closer than *D* = *2* m. Dots are the average by position and error bars are the corresponding standard deviation. Note that, as suggested by Figure 2, forwards are players that accumulate more time close to their rivals.

In Figure 4, we plot the average of the aggregated times filtered by players' position. We observe that forwards are the players who accumulate more time ($|t_{aggr}^{forward}|=20.83$ min) with a rival closer than a distance *D*, followed by defenders.

On the other side, goalkeepers are the players having less contact with the opponents ($|t_{aggr}^{goalkeeper}|=0.93$ min). See Appendix 1 summarising all average values and standard deviations.

**Figure 5:**
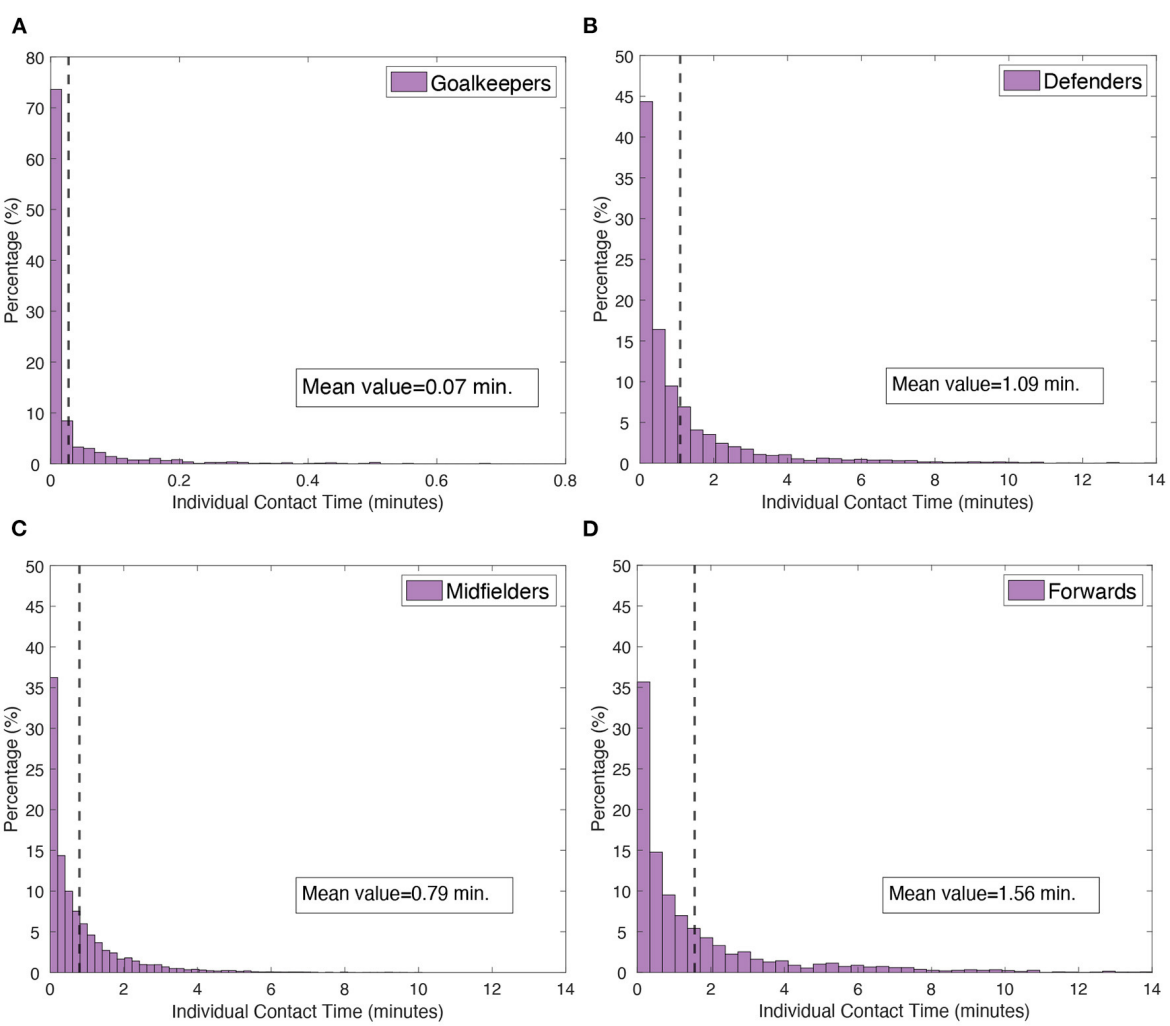
Individual contacts by player's position. Probability distribution functions (PDFs) of the accumulated time *t*_*accum*_ between pairs of players (player-player interaction), according to the position of each player: **(A)** goalkeepers, **(B)** defenders, **(C)** midfielders and **(D)** forwards. Note the difference in scale of the plot **(A)**. Contact time refers only to opponents. Dashed lines indicate the average value of the contact time of a player with an opponent, whose precise value is given in the box. The probability distribution functions show how heterogeneous the contacts are and, at the same time, how the peaks of all distributions are close to zero.

However, the heterogeneity of the contacts between players goes beyond the player position. Computing the accumulated time by pairs of players *t*_*accum*_ (instead of the aggregated one, considering all rival players) reveals high diversity in the contact time between players. Figure 5 shows the probability distribution function (PDF) describing the percentage of player-player interactions below (or equal) a distance *D*. Together with the PDFs, which are split into the four different player positions (in this case, we excluded substitutes), we indicated the average value of each class. Interestingly, all PDFs show similar behaviour, having a high peak very close to zero, which monotonically decreases as accumulated times increase. We can observe how the values of the probabilities related to goalkeepers are quite different from the rest of the players. On the one hand, the peak reaches values higher than 70%, indicating that goalkeepers' contact time is drastically lower than any other type of players.

This fact is also highlighted by the average contact time, which falls to $|t_{accum}^{goalkeeper}|=0.07\;$min. In the figure, the PDFs of defenders, midfielders and forwards have the same scale, however, the accumulated time of forwards' contacts ($|t_{accum}^{forward}|=1.56$ min) doubles the time of midfielders ($|t_{accum}^{goalkeeper}|=0.79$ min). The average value of defenders lies between ($|t_{accum}^{goalkeeper}|=1.09$ min). In any case, note that the highest probabilities are close to zero, and we observed that the majority of contacts between players correspond to accumulated times below 30 sec, no matter what the position of a player is.

**Figure 6:**
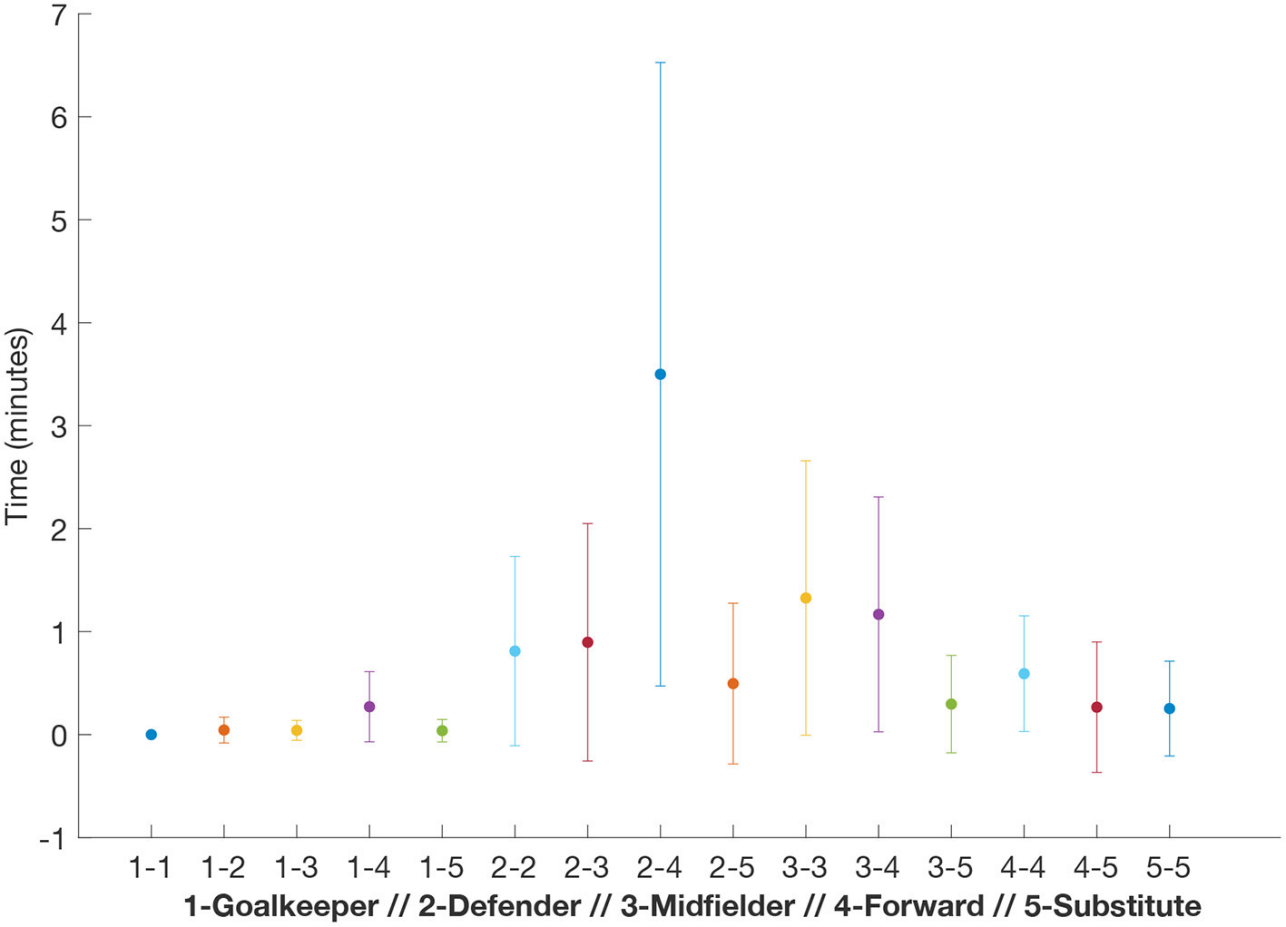
Interaction between player positions. Average contact time according to the position of a player and the opponent. Error bars correspond to the standard deviation. We can observe how the interactions between defenders and forwards accumulated the highest contact time, followed by the interactions between midfielders. On the other side, goalkeepers are those players that interact the least, with a contact time equal to zero with the opponent goalkeepers and the highest value with opponent forwards.

It is worth paying attention to the type of contact between players' position. In Figure 6, we focused on the positions of each pair of players intending to identify what kind of contacts are more frequent. In the figure, we can see how the contacts between defenders and forwards accumulate the highest average contact time. It is interesting to see that the standard deviation of these contacts is very high, indicating that, again, these contacts are very heterogeneous, which could be explained by the fact that each defender is prone to mark a specific forward and not all of them. Below the defender-forward interaction, we find the midfielder-midfielder and the midfielder-forward, both average values being slightly higher than 1 min. The rest of the contacts are below the minutes, with the goalkeeper-goalkeeper interaction being the lowest (zero, in fact), as one may have expected.

Finally, Table 1 summarises the time a player has been in contact (i) when the ball is in play and (ii) with players of the opponent team. Interestingly, only around 35% of the contacts are produced when the ball is in play. Concerning the contact with the rivals, it is only, on average, around 37% of the total contact time, i.e., players are more in contact with teammates. Interestingly, this behaviour is not fulfilled by goalkeepers who are around 68% of the time in contact with rival players. However, as we have shown before, the time accumulated by goalkeepers is much lower than the rest of the positions.

**Table 1:** Type of contact: ball in play and rivals.

	**Average (%)**	**Goalkeepers (%)**	**Defenders (%)**	**Midfielders (%)**	**Forwards (%)**	**Substitutes (%)**
Ball in play	35.70	17.56	36.64	39.12	39.57	40.53
Contact with rivals	37.20	68.35	40.49	42.57	31.13	35.66

*First row: Percentage of contact time with the ball in play (average and player position). Second row: Percentage of contact time with rivals*.

## Discussion

Distances between players during a soccer match are crucial to understanding phenomena as diverse as the risk of contagion between players or the team's organisation during the different phases of a match. Here, we have analysed players' distances with rivals, quantifying the player-player interaction time and the aggregated time with all opponents. Our results indicate that heterogeneity is one of the fundamental properties of the proximity between players. As we have seen, there is a high variation in the times accumulated by a player at distances to his rivals below a certain threshold. This behaviour is independent of the player position in the team and holds over all matches. When the focus is put on the player-player interactions, the analysis also uncovers a high heterogeneity. While the average distance to the rest of the rivals is below 2 min, it is more informative to pay attention to the probability distributions, showing that most of the interactions have a high peak close to zero. Importantly, in accordance with (Gonçalves et al., 2020), player-player interactions within 2 m were, in all cases, below 15 min, which is an indicator of low-risk exposure.

Although all players share common general properties, we also identified a series of differences in the distribution of proximity times depending on the players' position. Goalkeepers are the players who spend less time close to their rivals. Specifically, they accumulate <1 min per match with a rival closer than 2 m. In turn, their main interactions are with the forwards of their rival team. On the other hand, forwards are the players who stay closer to their opponents, with an average aggregated value of around 21 min. However, the player-player interactions of forwards have an average value lower than 2 min, again indicating a low-risk exposure.

Concerning the type of player-player interactions, we found that the defender-forward is the one leading by far the rest of possible combinations. At the same time, the standard deviation of this particular kind of interaction is very high, indicating a high heterogeneity again.

It is worth mentioning that most of the interactions are produced when the ball is not in play. This fact could be used to design efficient strategies to reduce the proximity times between players. Despite there are situations where it is not possible to constrain the distance between players when the ball is stopped, such as the preparation of corners or fouls, there are another, such as goal celebrations or referee complaints, which could be restricted for the shake of reducing the contact time between players.

Our results also have several implications in the context of the tactical analysis of soccer teams. On the one hand, the fact that the distancing between players is so heterogeneous makes reasonable the identification of those players with the highest proximity values. The fact that offensive and defensive phases can be identified with the tracking datasets, will allow to determine what defenders are covering rival players during more time and matrices as the one depicted in Figure 2 could help to understand how the marking of a given player is shared by two or more defenders, since individual marking relies on the proximity to the covered player. On that sense, Figure 4 shows an interesting result: Forwards are the players that spend the most time close to their rivals while midfielders are the field players (excluding goalkeepers) with the least accumulated time. This result indicates that forward players are those covered the most by their rivals in order to reduce their opportunities of creating danger. When looking at the specific interactions between the player positions, we clearly identify the defender-forward contact time to be the highest of all possible combinations (see Figure 6). This is somehow expected since, as mentioned before, individual marking of forward players is crucial to reduce their ability of movement and generating risky situations. However, it is worth mentioning that the three interactions accumulating the most time after the defender-forward interaction involve a midfielder player. In this way, midfielder-midfielder, midfielder-forward and midfielder-defender are ranked in the second, third and fourth position, respectively (see Figure 6). Therefore, while defenders are mainly concerned about covering forward players, midfielders have to mark players with different roles, a fact that may be taken into account in the preparation and training session.

Finally, all results shown in this paper refer to distances closer (or equal) than *D* = *2* m. However, qualitatively similar distributions are obtained at distances of *D* = *1.5* m and *D* = *1* metre, with the difference that the average accumulated times are lower in all categories since the value of *D* is decreased.

Concerning the limitations of our study, we did not have enough matches to adequately test different factors that may affect distancing between players (Lupo and Tessitore, 2016). One of them is the influence of the score, which has been demonstrated to influence different performance patterns of soccer teams (Lago-Peñas and Gómez-López, 2014). Another relevant performance indicator is the position of the team in the competition raking. The fact that we are just considering two fixtures at the middle of the season does not allow to track the evolution of teams in the raking and its relationship with the distancing between players. We expect that both variables, score and raking position, could be related to the average team distancing.

In view of all, we believe that these results should be taken into consideration when developing epidemiological models describing the risk of contagion during a soccer match (Buldú et al., 2020a; Gonçalves et al., 2020) but also to the understanding of the tactical performance of soccer teams.

## Conclusions

In this work we investigated what are the distances between players during a soccer match and how they depend on the role of a player in a team. We observed that distancing between players is highly heterogeneous as indicated by the accumulated time of players closer to a distance *D* = *2* to any other player of the match. We observed how the accumulated time close to a rival player is strongly influenced by the playing position. The individual distribution of time shows that forward players are at the top of the ranking and goalkeepers at its tail. When calculating the average accumulated time per position, forwards are those players accumulating the highest average time close to rivals, while goalkeepers are the ones with the least accumulated time. Concerning the specific player-player interactions, the defender-forward leads the ranking. The reported heterogeneity of the distancing between players can be crucial in the development of epidemiological models describing the risk of contagion during a match and in the interpretation of tactical aspects where the location and distancing between players plays a certain role.

## Data Availability Statement

The datasets presented in this article are not readily available because Datasets are owned by LaLiga. Requests to access the datasets should be directed to javier.buldu@urjc.es.

## Author Contributions

All authors participated in the conception of the article. RC and RR provided the datasets. DG, DA, and JB carried out the analysis. JB wrote the initial draught. All authors revised the manuscript together and gave final approval for publication.

## Conflict of Interest

The authors declare that the research was conducted in the absence of any commercial or financial relationships that could be construed as a potential conflict of interest.

## Publisher's Note

All claims expressed in this article are solely those of the authors and do not necessarily represent those of their affiliated organizations, or those of the publisher, the editors and the reviewers. Any product that may be evaluated in this article, or claim that may be made by its manufacturer, is not guaranteed or endorsed by the publisher.

## Appendix 1

**TABLE A1:** Summarises the average aggregated times (and corresponding standard deviations) depending on the players' positions shown in Figure 3. The average aggerated time |*t*_*aggr*_| consist of the total time accumulated by a player at a distance lower or equal than *D = 2* mto all his rivals.

	**Goalkeepers**	**Defenders**	**Midfielders**	**Forwards**	**Substitutes**
|*t*_*aggr*_| (minutes)	0.93 ± 0.73	14.64 ± 5.56	12.21 ± 4.74	20.83 ± 7.92	4.46 ± 3.45

***Table A1**. Aggregated times by position. Total time accumulated by a player at a distance lower or equal than D = 2 m to all his rivals, by position*.
